# Changes in perceived uselessness and risks for mortality: evidence from a National sample of older adults in China

**DOI:** 10.1186/s12889-017-4479-1

**Published:** 2017-06-09

**Authors:** Yuan Zhao, Matthew E. Dupre, Li Qiu, Danan Gu

**Affiliations:** 1 International Center for Aging and Health, Ginling College and School of Geography Science, Nanjing Normal University, Nanjing, China; 2 Duke Clinical Research Institute & Department of Sociology, Duke University, Durham, NC USA; 3 New York, New York, NY USA; 4 United Nations Population Division, Two UN Plaza, DC2, New York, NY -1910 USA

**Keywords:** Perceived uselessness, Self-perceptions of aging, China, Older adults, Oldest-old, Young-old

## Abstract

**Background:**

Self-perception of uselessness is associated with increased mortality risk in older adults. However, it is unknown whether and to what extent changes in perceived uselessness are associated with mortality risk.

**Methods:**

Using four waves of national longitudinal data of older adults from China (2005, 2008, 2011, and 2014), this study examines the association between changes in perceived uselessness and risk of subsequent mortality. Perceived uselessness is classified into three major categories: high levels (always/often), moderate levels (sometimes), and low levels (seldom/never). Five categories are used to measure change over three-year intervals: (1) persistently high levels, (2) increases to moderate/high levels, (3) persistent moderate levels, (4) decreases to moderate/low levels, and (5) persistently low levels. Cox proportional hazard models were used to estimate mortality risk associated with changes in levels of perceived uselessness.

**Results:**

Compared to those with persistently low levels of perceived uselessness, those with persistently high levels of feeling useless had 80% increased hazard ratio (HR) in mortality [HR =1.80, 95% CIs: 1.57–2.08, *p* < 0.001]; and those with increasing levels, persistently moderate levels, and decreasing levels of perceived uselessness had 42% [HR = 1.42, 95% CIs: 1.27–159, *p* < 0.001], 50% [HR = 1.50, 95% CIs: 1.32–1.71, *p* < 0.001], and 23% [HR = 1.23, 95% CIs: 1.09–1.37, *p* < 0.001] increased hazard ratio in mortality, respectively, when background characteristics were taken into account. The associations were partially attenuated when socioeconomic, family/social support, behavioral, and health-related covariates were individually taken into account. Older adults with persistently high and moderate levels of perceived uselessness still exhibited significantly higher risks of mortality (16% [HR = 1.16, 95% CIs: 1.00–1.135, *p* < 0.05] and 22% [HR = 1.16, 95% CIs: 1.06–1.139, *p* < 0.015], respectively) after adjusting for all covariates, although no significant mortality risks were found for either increasing to moderate/high levels or decreasing to moderate/low levels of perceived uselessness.

**Conclusions:**

Persistently high and moderate levels of perceived uselessness are associated with significant increases in mortality risk. These findings have important implications for promoting successful aging in China.

## Background

The self-perception of uselessness, a key component of one’s self-perception of aging, is the internalized feeling of one’s declining contribution and importance to others [1, 2]. Recent studies have shown that self-perceived uselessness is a negative psychological disposition that is significantly associated with higher mortality risks in older adults [1–10]. To date, however, much of the existing research is from developed counties and few studies consider how changes in perceptions of uselessness may impact mortality.

China is a developing country with a rapidly aging population and limited system of institutionalized care for its older adults [11]. With among the largest number of older adults in the world, China has gained considerable attention by scholars to better understand the challenges of aging in the context of this nation’s unique social and cultural makeup. Recent studies have now begun to demonstrate an association between perceptions of uselessness and subsequent health and mortality outcomes among older adults in China [11–13]. The research shows that older adults with high perceptions of feeling useless are more likely to exhibit poorer health and higher mortality risks than older adults with low (or no) perceptions of feeling useless [11–13]. However, it is unclear whether and to what extent changes in perceived uselessness that may accompany aging are associated with mortality risks. To our knowledge, only one study has shown that short-term changes in self-perceptions of aging are associated with subsequent mortality in U.S. adults aged 70–79 [2].

The current study is the first to use national longitudinal data of older adults (aged 65+) from China to examine the association between changes in perceived uselessness and subsequent mortality risks from 2005 to 2014. In addition, we examined whether factors related to (i) socioeconomic resources, (ii) family and support environment, (iii) health behaviors and lifestyle, and/or (iv) psychological and physical health status may contribute to the associations. The implications of these findings are discussed in the context of promoting successful aging in China.

## Methods

### Study sample

This study uses data from the 2005, 2008–2009, 2011–2012, and 2014 waves of the Chinese Longitudinal Healthy Longevity Survey (CLHLS). The first three waves of the CLHLS (1998, 2000, and 2002) were not included in this analysis because the 1998 and 2002 waves either did not include respondents aged 65–79 years, or the measures of self-perceived uselessness and social support were not consistent with subsequent waves. The CLHLS is an ongoing study conducted in a randomly selected half of the counties/cities in 22 provinces where Han is the major ethnicity. Nine minority-dominated provinces were excluded to avoid inaccurate age-reporting at very old ages (e.g., ages 90+) among ethnic minorities [14]. The total population of the 22 included provinces accounted for approximately 82% of the entire population of China in 2010. Additional details of the sampling procedures and assessments of data quality for the CLHLS are documented extensively elsewhere [14].

**Fig. 1:**
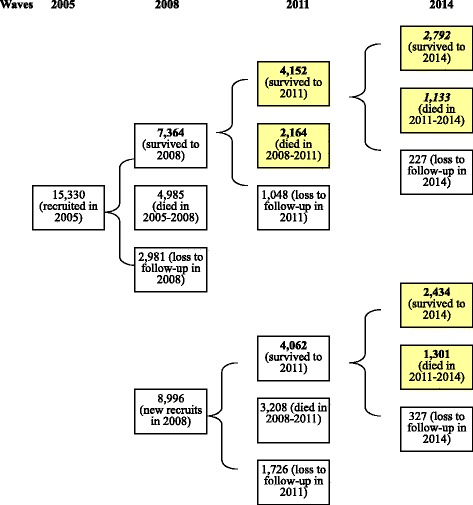
Construction of study sample by wave. Respondents who had at least two interviews were included in the calculation of changes in self-perceived uselessness (indicated with bold font). Respondents who had at least one episode of mortality risk were included in the study (indicated with shadowed font). Respondents who had three episodes of change in self-perceived uselessness and two episodes of mortality risk are indicated with italic font in the shadowed boxes. The total number of individuals included in the study is 10,051 (= 4152 + 2164 + 2434 + 1301) who contributed 13,976 observations (= 10,051 + 2792 + 1133)

An overview of the analytic sample for the present study is shown in Fig. 1. Note that only study participants who had at least two interviews from 2005 to 2014 were included in the analysis; and responses to the question of perceived uselessness in two time points (i.e., two adjacent waves) were used to identify changes over a three-year period. Subsequent mortality risks were determined by the respondents’ known survival status in waves 2008 to 2014. The final analytical sample for this study consisted of multiple longitudinal subsamples of respondents (*n* = 10,051 [=4152 + 2164 + 2375 + 1254]) who contributed 13,976 (10,051 + 2792 + 1133) observations from 2008 to 2014. The CLHLS subsamples were pooled together to improve the robustness of the mortality estimates.

### Measurement

#### Changes in perceived uselessness

Perceived uselessness was measured by the question "with age, do you feel more useless?" and included the following response categories: always, often, sometimes, seldom, almost never or never, and unable to answer. The responses were subsequently grouped to indicate three levels of perceived uselessness: always/often (high level), sometimes (moderate level), seldom/never (low level). Approximately 15% of participants selected “unable to answer” and roughly 90% of these cases were attributable to poor health [12], and especially cognitive impairment. Consistent with previous research, an additional category for those unable to answer was also included in the analyses to account for missing data in the models [13].

Changes in perceived uselessness were based on responses of perceived uselessness at two data points over a three-year interval: (1) persistently high levels (abbreviated as “high-high”), (2) increases from a lower level to a higher level (i.e., from a low level to a moderate/high level; or from a moderate level to a high level) (abbreviated as “increase to moderate/high”), (3) persistently moderate levels (“moderate-moderate”), (4) decreases from a higher level to a lower level (i.e., from a high level to a moderate/low level; or from a moderate level to a low level) (“decrease to moderate/low”), and (5) persistently low levels (“low-low”).

#### Mortality risk

All-cause mortality risk was ascertained by the respondents’ duration of exposure (in days) from the date of the 2008 interview until death (for the decedents) or to the date of the 2014 interview (for survivors). The dates of death were collected from official death certificates when available; otherwise, they were collected from the next-of-kin of the deceased respondents and local residential committees. The mortality data in the CLHLS was previously shown to be of high quality [14].

#### Covariates

The analyses included a wide range of covariates that have been previously linked to perceived uselessness and/or mortality [1, 2, 4, 11–13, 15–20]. All models adjusted for background characteristics and included age (categorized as 65–79, 80–89, 90–99, and 100+ to account for nonlinearity), sex, residence (urban vs. rural), and ethnicity (Han vs. non-Han). We also examined a number of factors related to (i) socioeconomic resources, (ii) family and support environment, (iii) health behaviors and lifestyle, and/or (iv) psychological and physical health status. Socioeconomic resources included years of formal schooling (0, 1–6, and 7+ years), primary lifetime occupation (professional/administration occupation vs. other occupation), economic independence (having a retirement wage or pension and/or own earnings vs. no), and family economic condition (rich/very rich vs. others).

Measures for family and support environment included currently married (yes vs. no), most frequent contact person (immediate family member, friend or other family member, or nobody), and co-residence with adult children (yes vs. no). Based on previous studies, measures of family support and environment also included (i) concordance/discordance between expected living arrangement and actual living arrangement and (ii) intergenerational transfers [13]. First, if an older adult was living alone or with a spouse—and was consistent with his/her expected living arrangement—we categorized them as concordant in living alone or with spouse. If an older adult was co-residing with children—and was consistent with his/her expected living arrangement—we categorized them as concordant in co-residence with children. All other older adults were categorized as either in a discordant living arrangement or currently institutionalized. In terms of intergenerational transfers, we categorized respondents according to whether they received money/food from children (yes vs. no) and whether they provided money/food to children (yes vs. no).

Health behaviors and lifestyle included current smoking (yes vs. no), current alcohol consumption (yes vs. no), regular exercise (yes vs. no), and the frequency of leisure activities and social participation. Levels of leisure activities were ascertained from six items: doing housework, gardening, raising domestic animals or poultry, reading books/newspapers, watching TV/listening to radio, and any other personal outdoor activities. Each item was measured on a five-point Likert scale (from never to almost daily) and the values were summed and categorized into tertiles to denote low, moderate, and high levels of leisure-time activity. Next, social participation was measured by two questions "do you participate in social activities?" and “do you play cards/mah-jong?” Following previous research, we categorized respondents into three groups of social participation: low level (never involved in these activities), high level (involved 1–7 times per week in at least one activity), and moderate level (the remaining respondents) [13].

Finally, psychological and physical health status included five major domains: activities of daily living (ADL), instrumental activities of daily living (IADL), chronic disease conditions, cognitive function, and psychological disposition. The measure for ADL disability included the self-reported ability to perform six daily activities—(a) bathing, (b) dressing, (c) indoor transferring, (d) toileting, (e) eating, and (f) continence. Following convention [10], respondents were dichotomized into those needing assistance in any one of the six tasks (ADL disabled) versus those needing no assistance in any of the six tasks (not ADL disabled). The measure for IADL disability included the self-reported ability to perform eight activities—(a) visiting neighbors, (b) shopping, (c) cooking, (d) washing clothes, (e) walking one kilometer, (f) lifting 5 kg, (g) crouching and standing up three times, and (h) taking public transportation. Consistent with ADLs, we dichotomized respondents into those needing help in any of the activities (IADL disabled) versus those needing no help in performing any of the activities (not IADL disabled). Chronic disease conditions were dichotomized into whether respondents reported any disease at the time of survey (from a list of more than twenty conditions [e.g., hypertension, heart diseases, stroke, diabetes, cancer, etc.]) versus no reported conditions. Fewer than 5% of the respondents reported two or more chronic conditions and the overall prevalence of conditions is comparable to those found in other national surveys in China [14]. Cognitive function was measured by a validated Chinese version of the Mini-mental Status Examination (MMSE), which included six domains of cognition (orientation, reaction, calculation, short memory, naming, and language) with a total possible score of 30 [14]. Respondents were dichotomized into being cognitively impaired (scores <24) or unimpaired (scores 24–30). Finally, psychological disposition was measured with four questions: "do you feel as happy as you did when you were younger?" (always/often vs. sometimes/seldom/never); “do you feel lonely?” (always/often vs. sometimes/seldom/never); "do you look at the bright side of things?" (always/often vs. sometimes/seldom/never); and "do you have control over things that happen to you?" (always/often vs. sometimes/seldom/never).

### Analytical strategy

Although preliminary analyses indicated that several factors (e.g., sex, ethnicity, education, etc.) violated the proportional hazard assumption for the bivariate Cox hazard models, these variables did not violate the proportional hazard assumption when all covariates were included in the analysis (results not shown). Therefore, the Cox proportional hazard models were used to estimate the relative hazard ratios (HR) for the association between perceived uselessness and mortality. The multivariate analyses were conducted in several steps. First, the association between changes in perceived uselessness and mortality was estimated while adjusting for the respondents’ background characteristics. Next, four sets of covariates were included in Models II through V to assess whether and to what extent they contributed to the association between perceived uselessness and mortality. The final step of analysis (Model VI) included all sets of covariates in a fully adjusted model.

Missing values on all variables in the analysis were less than 2%. We used the mode to impute the missing values for binary or categorical variables and the mean for continuous variables. Alternative imputation approaches were also assessed (e.g., regression-based imputations) and produced results that were nearly identical. Sampling weights were not used in the multivariate models because the CLHLS weights were constructed from population distributions of age, sex, and urban/rural residence. Research shows that standard errors are inflated when regression models include variables related to sample selection (i.e., age, sex, and urbanicity) [21]. Multi-collinearity was assessed in preliminary analyses. Results showed that values for the variance inflation factors for all of the covariates were less than 3; therefore, we concluded that there was no serious collinearity problem [22]. We also tested the sensitivity of the category “unable to answer” due to cognition on the outcomes. The results indicated that “unable to answer” had minimal impacts on the association between self-perceived useless and mortality risk. All analyses were performed using Stata version 13.1 [23].

## Results

The sample distributions of the study variables are presented in Table 1. The upper-left panel of the table shows the distribution of changes in perceived uselessness over time in the CLHLS. More than one-quarter of the respondents (27.0%) had an increase in their level of feeling useless—changing from low to moderate levels or from moderate to high levels. Alternatively, we find that slightly less than one-quarter of the respondents (24.2%) reported a decline in their level of feeling uselessness over time—changing from high to moderate levels or from moderate to low levels. Approximately 6.5%, 10.7%, and 15.4% of respondents had persistent levels of high, moderate, and low perceptions of uselessness, respectively, during the three-year interval. Roughly 16.2% of respondents were unable to answer (i.e., having missing information on this measure; 90% was due to poor health, primarily cognitive impairment). With regard to subsequent mortality risks, CLHLS respondents with persistently high levels of perceived uselessness had the highest rate of dying (38.3%) during the three-year interval. Conversely, those with persistently low levels of perceived uselessness had the lowest rate of death (19.6%).

**Table 1:** Sample distributions of study variables for the period 2005–2011, CLHLS

Variables	Sample %	Death %
Total (*n*)	13,976	33.1
Changes in perceived uselessness		
High-high	6.4	38.3
Low-moderate/moderate-high	27.0	30.1
Moderate-moderate	10.7	31.7
High-moderate / moderate-low	24.2	26.2
Low-low	15.4	19.6
High/moderate/low- unable to answer	8.1	62.8
Unable to answer - high/moderate/low	5.8	45.8
Persistently unable to answer	2.4	75.3
Covariates		
*Background Characteristics*		
Mean age (years)	85.1	−
Ages 65–79^a^	36.0	10.9
Ages 80–89^a^	27.6	28.6
Ages 90–99^a^	25.2	53.3
Ages 100+^a^	11.1	69.4
Females	54.6	34.3
Males	45.4	31.5
Non-Han ethnicity	11.0	29.5
Han ethnicity	89.0	33.3
*Socioeconomic Resources*		
Education, 0 year of schooling	59.9	37.4
Education, 1–6 years of schooling	29.5	27.3
Education, 7+ years of schooling	10.6	23.2
Rural	52.9	33.8
Urban	47.1	32.0
Non-White collar occupation	91.8	33.6
White collar occupation	8.2	25.4
Economic dependence	71.3	38.3
Economic independence	28.7	19.5
Fair or poor family economic condition	84.7	33.6
Rich family economic condition	15.3	29.3
*Family and Support Environment*		
Currently not married	62.1	41.6
Currently married	37.9	18.8
Family members are most frequent contacts	75.6	32.0
Friends/relatives are most frequent contacts	18.0	27.7
No one to contact	6.4	58.7
No coresidence with children	43.1	24.0
Coresidence with children	56.9	39.6
Discordance in living arrangement	26.4	35.6
Concordance in living alone/with spouse only	30.1	20.3
Concordance in coresidence with children	43.5	39.9
Not-receiving money/food from children	21.5	33.0
Receiving money/food from children	78.5	32.9
Not-giving money/food to children	76.4	35.8
Giving money/food to children	23.6	23.6
*Health Behaviors and Lifestyle*		
Not currently smoking	81.9	34.5
Currently smoking	18.1	25.3
No current alcohol consumption	82.8	34.2
Current alcohol consumption	17.2	26.8
No regular exercise	66.0	39.0
Regular exercise	34.0	21.0
Leisure activity (low level)	29.7	61.2
Leisure activity (medium level)	30.9	29.2
Leisure activity (high level)	39.4	14.4
Social participation (low level)	75.3	38.2
Social participation (medium level)	10.6	18.3
Social participation (high level)	14.1	15.6
*Psychological and Physical Health Status*		
ADL independent	77.3	24.6
ADL dependent	22.7	61.1
IADL independent	36.4	11.6
IADL dependent	63.6	45.1
Cognitively unimpaired	63.7	20.3
Cognitively impaired	36.3	55.0
Having no chronic disease	39.3	33.8
Having 1+ chronic disease	60.7	32.3
Not often as joyful as when younger	64.2	35.9
Often as joyful as when younger	35.8	27.5
Not often lonely	93.1	32.5
Often lonely	6.9	38.2
Not optimistic	24.3	37.8
Optimistic	75.7	31.3
No self-control	41.7	42.7
Self-control	58.3	25.9

Note: (1) Values are based on 13,976 observations (from 10,051 individuals interviewed from 2005 to 2014 [refer to Fig. 1]); (2) Death % refers to deaths occurring in 2008–2011 or 2011–2014 among 9888 individuals; (3) Results are unweighted

^a^Age at the beginning of the survey interval 2008–2011 or 2011–2014

Table 2 reports the HRs of mortality risk associated with changes in perceived uselessness for the CLHLS sample of older adults in China. Results show that mortality risks were higher in those with consistently high levels of perceived uselessness [HR = 1.80, 95% CIs: 1.57–2.08, *p* < 0.001], increases to moderate/high levels [HR = 1.42, 95% CIs: 1.27–159, *p* < 0.001], consistently moderate levels [HR = 1.50, 95% CIs: 1.32–1.71, *p* < 0.001], and decreases to moderate/low levels [HR = 1.23, 95% CIs: 1.09–1.37] compared with those reporting consistently low levels of perceived uselessness when adjusting for background demographics (Model I). The associations are only slightly attenuated when socioeconomic resources (Model II) and family/support environment (Model III) were further taken into account. The associations were reduced to a greater extent when adjusting for behavioral and lifestyle factors (Mode IV)—with no significant increase in mortality for those with decreases to moderate/low levels of perceived uselessness. As expected, psychological and physical health status further accounted for the associations between changes in perceived uselessness and mortality (Model V). When all sets of factors were included (Model VI), the associations between changes in perceived uselessness and mortality further diminished: only the persistently high [HR = 1.16, 95% CIs: 1.00–1.35, *p* < 0.05] and moderate [HR = 1.06, 95% CIs: 1.06–1.39, *p* < 0.01] groups had significantly higher risks of mortality.

**Table 2:** Relative risk (RR) of mortality of dynamic changes in self-perceived uselessness, CLHLS 2005–2014, Ages 65+, unweighted

	Model I	Model II	Model III	Model IV	Model V	Model VI
Changes in perceived uselessness						
High-high (vs. low-low)	1.80 *** (1.57–2.08)	1.68 *** (1.46–1.93)	1.73 *** (1.50–1.99)	1.39 *** (1.21–1.61)	1.26 ** (1.09–1.46)	1.16 * (1.00–1.35)
Low-moderate / moderate-high (vs. low-low)	1.42 *** (1.27–1.59)	1.35 *** (1.21–1.51)	1.40 *** (1.26–1.69)	1.22 *** (1.09–1.36)	1.12 * (1.00–1.25)	1.07 (0.95–1.20)
Moderate-moderate (vs. low-low)	1.50 *** (1.32–1.71)	1.44 *** (1.26–1.64)	1.48 *** (1.30–1.69)	1.33 *** (1.16–1.51)	1.27 *** (1.12–1.46)	1.22 ** (1.06–1.39)
High-moderate / moderate-low (vs. low-low)	1.23 *** (1.09–1.37)	1.18 ** (1.05–1.32)	1.21 ** (1.08–1.35)	1.10 (0.98–1.23)	1.08 (0.96–1.21)	1.03 (0.92–1.15)
Log pseudolikelihood	−41,087.8	−41,056.3	−41,020.5	−40,761.4	−40,701.5	−40,565.9

Note: Model I controlled for background characteristics (i.e., age, sex, ethnicity). Model II added socioeconomic resources (i.e., educational attainment, urban-rural residence, lifetime occupation, economic independence, and family economic conditions). Model III added family and support environment (i.e., marital status, frequently-contacted persons, concordance with children, concordance in living arrangement, intergenerational exchanges) to Model I. Model IV added health behaviors and lifestyle (smoking, alcohol consumption, regular exercise, leisure activities, and social participation) to Model I. Model V added psychological and physical health conditions (ADL and IADL disabilities, cognitive impairment, chronic disease conditions, and psychological well-being) to Model I. Model VI included all covariates

*p < 0.05, **p < 0.01, ***p < 0.001

## Discussion

Perceived uselessness has received increasing attention in the literature and has been linked to significant increases in mortality [1–11]. Although self-perceptions of aging (and usefulness) are generally internalized over the life course, recent research has shown that changes in perceptions of uselessness are common in older adults [2]. Using a uniquely large nationally representative longitudinal dataset of older adults in China, we found that persistently high levels of perceived uselessness were associated with increased mortality risk; whereas persistently low levels of perceived uselessness was associated with reduced mortality risk. Controlling for more than two dozen socioeconomic, social support, behavioral, and health-related factors accounted for some, but not all of the associations.

Approximately 60% of CLHLS respondents reported changes in the frequency of feeling useless over a three-year interval—a rate that is somewhat higher than reported in the United States (40%) [2]. Our data also showed that the proportions of study participants reporting changes in these perceptions were similar in direction (27% increase and 24% decrease) and consistent with U.S. research [2]. Studies suggest that older adults in China often attribute more weight to family dynamics in rating the subjective quality of their aging [24], and that interrelated changes in living arrangements [25, 26] and health [27] are increasingly common at old age. In this context, older adults may change their perceptions of usefulness to family members and others as these events occur over the latter life course. In addition, older adults’ feelings of uselessness may be influenced by the migration of children (for employment opportunities) and relocation/resettlement of children due to the rapid urbanization of both urban and rural areas in China. Future research is needed to further examine these and other factors associated with such changes in perceived uselessness in older adults.

To date, only one U.S. study has examined the association between changes in feelings of usefulness and mortality. Consistent with our findings, results from U.S. data show that persistently high levels of feeling useful to others was associated with significantly lower risk of mortality [2]. In China, there is also evidence to suggest a significant association between perceived uselessness and mortality in older adults [11]. The current study builds upon this literature by demonstrating an association between changes in perceived uselessness and mortality in a major developing country. In the context of China, resources for successful aging are limited compared with Western societies; and perceptions of aging are deeply rooted in cultural norms and expectations [13, 28]. Our findings show that persistently high levels of feeling useless to others was associated with an 80% increased hazard ratio in mortality compared with those who report consistently low levels of feeling useless. Although adjustments for multiple covariates accounted for much of the association, older adults with high levels of perceived uselessness remain at elevated risk for mortality relative to their counterparts with low levels of perceived uselessness.

Existing literature suggests that older adults who frequently report high levels of uselessness often possess fewer social connections, lower self-efficacy and control, less social support, and lower levels of resilience and capacity compared with those who do not perceive themselves as useless [1, 3]. Furthermore, there is evidence to indicate that perceived uselessness is also associated with engagement in fewer social activities [29] and health-seeking behaviors [30], which in turn, may precipitate or exacerbate health problems [31]. From a physiological standpoint, exhibiting strong feelings of uselessness may cause dysregulation of the central nervous system, neurotransmitters, and/or immune system that may lead to the onset and progression of disease, disability, and other manifestations of aging [32, 33]. Alternatively, positive feelings toward one’s aging and usefulness to others may promote positive lifestyles such as a healthy diet, routine medical check-ups, exercise, and more leisure-time activities that fulfill their expected social roles [1]. In sum, older adults’ perceptions of usefulness or uselessness may impact their health through psychological, behavioral, and/or physiological pathways [19, 20, 25].

Major strengths of the present study include its large sample size, longitudinal design, and the nationally representativeness of a non-Western society that includes more than 10,000 older adults observed over a 9-year period. Our findings also have potential implications for public health. The robust associations between high and moderate levels of uselessness and mortality suggest that older adults’ perceptions may have a direct impact on trajectories of health and longevity. These findings underscore the importance of maintaining positive self-perceptions with age and that it may never be too late to promote positive perspectives of aging. Studies show that older adults’ perception of usefulness may be influenced by the public [34, 35]; therefore, public health interventions may consider promoting positive views on aging that target not only older adults, but also in the context of families, neighborhoods, and society [1, 19, 33, 36]. Given recent evidence on the negative perceptions of aging among older adults in China [37], such strategies are especially timely and needed to promote more successful aging.

Several limitations of the study are also acknowledged. First, we recognize that perceived uselessness was measured by a single question in the CLHLS, which may have measurement bias. Therefore, we encourage additional studies to further substantiate these findings using more sophisticated and multi-domain measures to better capture the complex nature of self-perceptions of aging and uselessness [4, 11]. Second, we acknowledge that data were lacking on the length of self-perceived uselessness and the timing of changes in self-perceived uselessness. It is unclear whether and to what extend such individual variations would alter the results; therefore, more research is warranted to shed light on these dimensions perceived uselessness. Third, this study did not examine the underlying factors that may be contributing to changes in these perceptions. More research is needed to identify such factors—particularly those related to increased feelings of uselessness. Fourth, we did not investigate potential differences in the associations among subgroups (e.g., by age, urban/rural residence, etc.). We recognize that some segments of the population may internalize different perceptions of aging and usefulness [13] and the subsequent link to mortality may not be universal. Therefore, we encourage future studies to consider possible subgroup variations in these findings—which may provide additional insights into how feelings of uselessness impact mortality [11]. More generally, more empirical work is needed to better understand how self-perceptions of aging and one’s sense of usefulness influence survival in China and other nations.

## Conclusions

In sum, our study provides the first evidence of a significant association between persistent and changing perceptions of uselessness and mortality in a traditionally Confucian country. Unlike many Western countries, China’s culture has a strong tradition of respect for elders and a deeply-rooted culture that emphasizes family support, filial piety, and family harmony [24, 38, 39]. The results from this study broaden our understanding of how internalized perceptions of aging can have significant consequences for the survival and longevity of older adults in a rapidly aging society.

## Abbreviations

ADL: Activities of daily living
CLHLS: Chinese Longitudinal Healthy Longevity Survey
HR: Hazard ratio
IADL: Instrumental activities of daily living
MMSE: Mini-mental status examination
